# Intrinsic cellular signaling mechanisms determine the sensitivity of cancer cells to virus-induced apoptosis

**DOI:** 10.1038/srep37213

**Published:** 2016-11-16

**Authors:** Yunfei Wang, Dawei Li, Jian Luo, Guimei Tian, Lisa Y. Zhao, Daiqing Liao

**Affiliations:** 1 Department of Anatomy and Cell Biology, UF Health Cancer Center, UF Genetics Institute, University of Florida College of Medicine, Gainesville, Florida, USA; 2 Shaanxi Key Laboratory of Agriculture Molecular Biology, Department of Biochemistry and Molecular Biology, College of Life Science, Northwest Agriculture and Forestry University, Yangling, Shaanxi, China; 3 Department of Urology, Qilu Hospital, Shandong University, Jinan, Shandong, China

## Abstract

Cancer cells of epithelial and mesenchymal phenotypes exhibit different sensitivities to apoptosis stimuli, but the mechanisms underlying this phenomenon remain partly understood. We constructed a novel recombinant adenovirus expressing Ad12 E1A (Ad-E1A12) that can strongly induce apoptosis. Ad-E1A12 infection of epithelial cancer cells displayed dramatic detachment and apoptosis, whereas cancer cells of mesenchymal phenotypes with metastatic propensity were markedly more resistant to this virus. Notably, forced detachment of epithelial cells did not further sensitize them to Ad-E1A12-induced apoptosis, suggesting that cell detachment is a consequence rather than the cause of Ad-E1A12-induced apoptosis. Ad-E1A12 increased phosphorylation of AKT1 and ribosomal protein S6 through independent mechanisms in different cell types. Ad-E1A12–induced AKT1 phosphorylation was PI3K-dependent in epithelial cancer cells, and mTOR-dependent in mesenchymal cancer cells. Epithelial cancer cells upon Ad-E1A12-induced detachment could not sustain AKT activation due to AKT1 degradation, but AKT1 activation was maintained in mesenchymal cancer cells. Expression of epithelial cell-restricted miR-200 family in mesenchymal cells limited mTOR signaling and sensitized them to Ad-E1A12-induced cell killing. Thus, epithelial cancer cells rely on the canonical PI3K-AKT signaling pathway for survival, while mesenchymal cancer cells deploy the PI3K-independent mTORC2-AKT axis in response to strong death stimuli.

The propensity to undergo apoptosis varies widely among diverse cancer cells. Attachment of epithelial cells to the extracellular matrix (ECM) is required for the maintenance of proper cellular polarity and tissue structure. ECM detachment of epithelial cells including carcinoma cells of epithelial phenotypes can trigger a form of cell death known as anoikis^1^. Studies on mammary epithelial cells demonstrate that ECM-deprived cells result in lysosome-mediated degradation of the epidermal growth factor receptor (EGFR) and downregulation of RTK-mediated cell survival signaling, leading to the upregulation of proapoptotic protein Bim and cell death^2^,^3^,^4^. This intrinsic apoptotic mechanism limits the survival of disseminated cancer cells and thus their distant metastatic colonization^5^,^6^. It has been estimated that less than 0.1% of spreading cancer cells survive the harsh stresses of infiltrating and colonizing distant organs. This selection process leads to a population of resilient cancer cells that can survive in the presence of powerful intrinsic and extrinsic death stimuli and withstand repeated cycles of therapies. A variety of mechanisms exist to protect disseminated cancer cells from anoikis^5^,^6^, among which growth factor receptor-mediated AKT activation seems to play a critical role^3^,^4^,^7^,^8^. Indeed, overexpression of ERBB2 (HER2/NEU) stabilizes EGFR and promotes the survival of ECM-deprived epithelial cells^2^, underscoring the importance of RTK-mediated signaling for anoikis resistance.

Epithelial cancer cells detached from native ECM may survive after successfully undergoing epithelial-mesenchymal transition (EMT) by engaging prosurvival factors through tumor cell-autonomous autocrine signaling or paracrine interactions within a specific microenvironment. The expression of several transcription factors including Snail, Slug, Twist, Zeb1 and Zeb2, as well as the downregulation of a number of microRNAs such as the miR-200 family underlie cancer cells with the mesenchymal phenotype^9^,^10^. The expression of EMT markers exhibits a clear inverse correlation with that of the miR-200 family as revealed in an analysis of the Cancer Genome Atlas data sets for breast and lung cancers^11^. Notably, miR-200c targets neurotrophic tyrosine receptor kinase type 2 (NTRK2 or TrkB)^12^ and its ligand neurotrophin 3 (NTF3)^13^. In mesenchymal cancer cells, increased expression of both TrkB and NTF3 as a result of miR-200c downregulation confers anoikis resistance^12^,^13^. High-level expression of the miR-200 family is observed in the breast cancer cells of epithelial morphology such as the cells of luminal breast cancer subtypes^10^. In contrast, breast cancer cells of mesenchymal phenotypes such as cells from the basal subtype generally express a low level of the miR-200 family^10^,^14^. Thus, complex genetic and epigenetic changes along with altered cellular signaling determine the fate of disseminated cancer cells.

Among the different breast cancer clinical subtypes, the triple-negative subtype that lacks the expression of hormone receptors (estrogen and progesterone receptors) and ERBB2 displays similar gene expression profiles and cell-biological features to the basal molecular subtype. Triple-negative breast cancer (TNBC) has a higher tendency to develop distant metastasis, resistance to therapy and disease recurrence^15^. Most TNBC cells are phenotypically mesenchymal-like, while cancer cells of the luminal subtypes, including the ERBB2-enriched subtype, have an epithelial appearance. Interestingly, these subtypes also show distinct gene mutational patterns^16^. For example, the mutation of *PIK3CA* encoding the p110α catalytic subunit of the class IA phosphatidylinositol 3-kinase (PI3K) has a much higher frequency in luminal subtypes (43%) compared to basal subtypes (7%), while the inverse is true for *TP53* mutations with 84% cases of basal subtypes carrying *TP53* mutations compared to 27% in luminal subtypes^16^. These findings suggest that different breast cancer subtypes depend on distinct cellular signaling pathways for survival and sustained proliferation.

The signaling pathways that determine differential sensitivity of epithelial and mesenchymal cancer cells to apoptosis remain incompletely understood. Previously, it was shown that the expression of Ad5 E1A 243R (the small E1A isoform) sensitizes apoptosis of epithelial cells whose interactions with the matrix are disrupted (anoikis) through trypsinization^17^. The ability of Ad5 E1A to induce the expression of genes that confer epithelial phenotype appears to promote anoikis^17^. In particular, the interaction between E1A and CtBP appears to be critical for anoikis sensitization, possibly by relieving Zeb1-mediated gene repression^18^. To facilitate the understanding of signaling pathways underlying differential sensitivity of different cancer cells to apoptosis, we have constructed a novel recombinant adenovirus (Ad-E1A12) that expresses the large E1A isoform of adenovirus type 12 (Ad12), which is a highly oncogenic serotype among >50 adenovirus species^19^,^20^. We report herein that cancer cells of epithelial and mesenchymal phenotypes display different sensitivity to Ad-E1A12. Ad-E1A12 triggered spontaneous cell detachment and apoptosis of tumor cells with epithelial phenotypes. In contrast, cancer cells with mesenchymal features are highly resistant to Ad-E1A12. Distinct cell survival signaling mechanisms appear to determine phenotypic outcomes in response to the strong apoptotic stimuli upon Ad-E1A12 infection.

## Results

### Construction of Ad-E1A12

**Figure 1:**
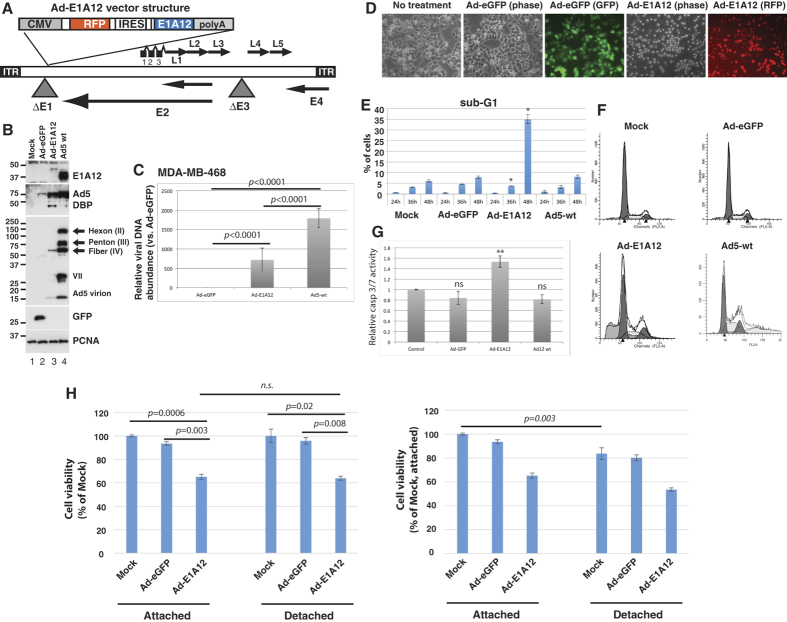
Ad-E1A12 induces spontaneous cell detachment and apoptosis. (**A**) The vector structure of Ad-E1A12. (**B**) Expression of E1A12, Ad5 DBP and viral capsids in MDA-MB-468 cells as determined by Western blotting. (**C**) Replication of the viral genome of Ad-eGFP (replication defective), Ad-E1A12 and wt Ad5 in MDA-MB-468 cells. The relative viral DNA abundance at 48hpi was determined by qPCR. (**D**) Spontaneous detachment of MDA-MB-468 cells upon Ad-E1A12 infection. Photomicrographs of uninfected MDA-MB-468 cells and those infected with Ad-eGFP, or Ad-E1A12. Fluorescent images of cell cultures infected with Ad-eGFP or Ad-E1A12 are also shown. (**E**) Induction of apoptosis in MDA-MB-468 cells by Ad-E1A12 as assessed by flow cytometry. The percentages of cells with sub-G1 DNA content are plotted. (**F**) Representative cell-cycle profiles of uninfected MDA-MB-468 cells and those infected with Ad-eGFP, Ad-E1A12, and Ad5 wt at 48hpi. (**G**) Caspase activation of MDA-MB-468 cells infected with Ad-E1A12. The caspase activity was assessed with the Caspase-Glo^®^ 3/7 assay system (Promega) in MDA-MB-468 cells without infection (control), infected in Ad-eGFP, Ad-E1A12, or wild-type Ad12 virus (Ad12 wt). *p < 0.05, **p < 0.01 (vs. control, Student’s t-test); ns: not statistically significant. H, Ad-E1A12-induced cell death is independent of cell detachment. MDA-MB-468 cells were uninfected, or infected with Ad-eGFP or Ad-E1A12. Cells were reseeded 4 h post-infection in a regular 96-well plate (“Attached”) or an ultra-low attachment 96-well plate (“Detached”). Cell viability assay was done using CellTiter-Glo at 48 h post-infection. The *p* values of pairwise comparison are shown based on Student’s t-test.

In the Ad-E1A12 vector, the expression cassette of the large E1A protein (266R) of Ad12 (E1A12) was inserted in the *E1* region of an Ad5 vector (Fig. 1A). This novel virus lacks the expression of anti-apoptotic E1B 19-kDa and 55-kDa proteins as well as the gene products of the *E3* region. In the breast cancer MDA-MB-468 cell line, Ad-E1A12 induced a high-level expression of the Ad5 *E2A* gene product DBP (DNA-binding protein) required for viral genome replication, although the yield of the viral genome and the expression of capsids were significantly impaired compared to Ad5 (Fig. 1B and C). Viral gene expression and genome replication were observed in diverse cancer cell lines (see Supplemental Fig. S1).

### Ad-E1A12 triggers spontaneous detachment and apoptosis of epithelial cancer cells

To assess the cell-biological effects of Ad-E1A12 that does not express viral anti-apoptotic proteins, we infected MDA-MB-468 cells with Ad-E1A12 or the control virus Ad-eGFP and analyzed cell cycle profiles of the infected cells. At 24 h post infection (pi), the cell cycle profiles of uninfected cells and those infected with Ad-eGFP or Ad-E1A12 were largely similar (data not shown). At 48hpi, cells with sub-G1 DNA content characteristic of apoptotic cells increased sharply in Ad-E1A12-infected cells, when compared with both uninfected control cells and those infected with Ad-eGFP or wt Ad5 (Fig. 1E and F). Morphologically, MDA-MB-468 cells infected with Ad-E1A12 became rounded, shrunken and completely detached from the culture plate, whereas uninfected or Ad-eGFP-infected cells maintained normal epithelial morphology (Fig. 1D). Caspase activity was elevated in Ad-E1A12-infected cells compared to those infected with Ad-eGFP or wt Ad12 (Fig. 1G). The data presented here indicate that Ad-E1A12 induces spontaneous detachment and apoptosis without the requirement of prior detachment of cells from the matrix, suggesting that E1A12 is a more powerful inducer of cell death. Similar spontaneous detachment was also observed in diverse epithelial cancer cell lines infected with Ad-E1A12 including HCT116 (colon) and LNCaP (prostate) cancer cells (Supplemental Fig. S2).

Previous studies have shown that the Ad5 E1A 243R protein could sensitize cells to anoikis^17^,^18^. We wished to test whether Ad-E1A12 also has this property. MDA-MB-468 cells were uninfected or infected with the indicated viruses. The cells were then seeded in a regular culture plate or an ultra-low attachment plate; cell viability was measured at 48 h post infection. Whereas an overall loss of viability was observed for MDA-MB-468 cells cultured in suspension under all tested conditions (Fig. 1H, right panel), detached cells was not more susceptible to Ad-E1A12-induced apoptosis (Fig. 1H, left panel). Similar results were observed at 24 h post infection, when cells infected with Ad-E1A12 were not yet detached (data not shown). These results indicate that while cell detachment contributes to the extent of apoptosis in cells infected with Ad-E1A12, detachment per se is not required for Ad-E1A12 to trigger apoptosis.

We have assessed the impact of Ad-E1A12 infections on cell viability of a panel of diverse cancer cell lines. Table 1 lists the EC_50_ values of Ad-E1A12 against various cancer cell lines of diverse tissue origins. Overall, it appears that cells displaying epithelial morphologies such as MDA-MB-468, MCF7, LNCaP, HPAC and BxPC3 are more susceptible to Ad-E1A12 infection than those with mesenchymal characteristics (MDA-MB-231, PC-3 and MIA PaCa-2). These mesenchymal cell lines failed to detach upon Ad-E1A12 infection. Collectively, these data indicate that Ad-E1A12 potently kills epithelial cancer cells through disrupting ECM attachment and apoptosis.

### Downregulation of anti-apoptotic proteins in epithelial cancer cells infected with Ad-E1A12

**Figure 2:**
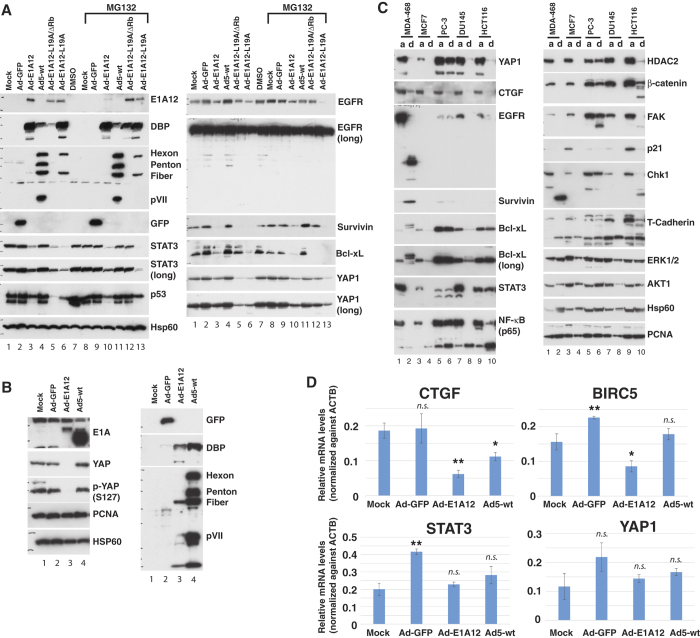
Protein degradation of matrix-detached cells. (**A**) MDA-MB-468 cells were uninfected or infected with the indicated viruses. The proteasomal inhibitor MG132 was added to 20 μM 6 h before cell lysis as indicated. Cells were lysed at 48hpi for Western blotting using antibodies against the indicated proteins. (**B**) Effects of Ad-E1A12 infection on YAP1 phosphorylation. MDA-MB-468 cells were treated as in panel (A). Western blotting was conducted with antibodies against the indicated proteins. Note that a different antibody was used from what was used in panel A for detecting E1A. This antibody also detected Ad5 E1A. (**C**) Downregulation of cellular proteins after detachment of cancer cells. The indicated cancer cell lines were trypsinized and then immediately lysed (d) or lysed directly on plate (a). The lysates were subjected to Western blotting with antibodies against the indicated proteins. (**D**) Quantitative real-time PCR (qRT-PCR) was done for the indicated genes using RNAs isolated from MDA-MB-468 cells that were mock treated or infected with the indicated viruses. Shown are average values (normalized against ACTB mRNA levels) of three biological replicates along with standard errors of the mean (error bars). *p < 0.05, **p < 0.01, *n.s.*: not specific (vs. mock, Student’s t-test).

To understand potential mechanisms underlying Ad-E1A12-induced apoptosis, we analyzed the expression of a number of proteins important for cell survival. As shown in Fig. 2A, EGFR, which is overexpressed in MDA-MB-468 cells, was clearly downregulated in cells infected with Ad-E1A12. Similarly, the pro-survival transcription factor STAT3, the highly expressed p53 R273H mutant, and the anti-apoptotic proteins Survivin and Bcl-xL were also drastically downregulated (Fig. 2A). E1A interacts with a host of cellular proteins including acetyltransferase/coactivator p300, retinoblastoma protein (RB), and CtBP, and these interactions determine functional properties of E1A^21^. We have constructed recombinant virus carrying a mutated E1A12 (E1A12-L19A, or E1A12-L19A/ΔRB), in order to test potential roles of these important interactions in E1A12-induced apoptosis. The L19A mutation likely attenuates interactions of E1A with a number of cellular proteins^22^,^23^, while the Rb-binding LxCxE motif was deleted in addition to the L19A mutation in E1A12-L19A/ΔRb mutant. These mutations did not affect Ad-E1A12-induced apoptosis (data not shown) and downregulation of STAT3 or p53 (Fig. 2A).

Treatment of the infected cells with the proteasomal inhibitor MG132 was largely ineffective in restoring the expression of these proteins (Fig. 2A), suggesting that their downregulation was not due to proteasomal degradation. Consistently, it was shown that detachment-induced EGFR degradation is mediated through the lysosome^2^,^3^,^4^. In contrast, cells infected with wt Ad5 did not induce the degradation of these proteins (Fig. 2A). Of note, the downregulation of STAT3, p53, Survivin, and YAP1 was inhibited by MG-132 in cells infected with Ad-E1A12- L19A/ΔRB (Fig. 2A lane 12).

The Hippo tumor suppressor pathway is activated upon cell detachment from ECM, resulting in the phosphorylation and inactivation of the Yes-associated protein (YAP1) transcription coactivator and anoikis^24^. In MDA-MB-468 cells infected with viruses expressing an E1A12 construct, YAP1 levels were clearly reduced when compared to uninfected cells or cells infected with Ad-GFP, or wt Ad5 (Fig. 2A), suggesting that the Hippo pathway may also be activated in cells infected with Ad-E1A12. Correspondingly, phosphorylated YAP1 was also undetectable (Fig. 2B). Quantitative real-time RT-PCR experiments showed that the corresponding mRNA levels for YAP1 and STAT3 were similar in MDA-MB-468 cells infected with Ad-E1A12 to those mock infected (Fig. 2D), indicating that their reduced expression was likely due to protein degradation in ECM-detached cells. Of note, the mRNA levels of the well-established endogenous YAP1 target genes CTGF and BIRC5 (encoding Survivin)^25^ were clearly reduced in cells infected with Ad-E1A12, compared to controls (mock and Ad-E1A12, Fig. 2D), indicating that the YAP1 transcriptional activity was also inactivated.

We then examined whether the observed downregulation of cell survival proteins in cells infected with Ad-E1A12 may be a direct consequence of cell detachment. We compared protein levels in cells that were lysed directly with a detergent-containing buffer (a) or detached after trypsin treatment (d). As shown in Fig. 2C, YAP1 was dramatically downregulated in the detached cells compared to attached cells in MDA-MB-468 and several other epithelial cancer cell lines including MCF7 (luminal breast cancer), DU145 (prostate cancer) and HCT116 (colon cancer) with the exception of the mesenchymal prostate cancer cell line PC-3, which is known to be resistant to anoikis^26^. Downregulation of the YAP1 target gene CTGF (connective tissue growth factor) and several other proteins such as STAT3, NF-κB, Chk1, β-catenin, HDAC2, focal adhesion protein kinase (FAK), and p21^CIP1^ (Fig. 2C) was also observed. In MDA-MB-468 cells detached by trypsin, the cell survival protein Survivin were upregulated (Fig. 2C, lane 2), which may serve to temporarily maintain viability in detached cells, as most cells remain viable immediately after trypsinization. Of note, several bands of the anti-apoptotic protein Bcl-xL with different molecular weights appeared in detached MDA-MB-231 cells (Fig. 2C). Remarkably, although EGFR downregulation was observed in Ad-E1A12-infected cells as well as in trypsin-detached cells, several smaller fragments of EGFR were detected in trypsin-detached cells but not in Ad-E1A12-infected cells (Fig. 2A and C). Because the antibody we used detects the C-terminal cytoplasmic domain of EGFR and the size of the major band is about 45-kDa, it might be the recently described EGFR splice variant called mLEEK that is overexpressed in tumors^27^. Alternatively, the small EGFR fragments could be due to the degradation of the extracellular domain of EGFR. By contrast, degradation of cell survival proteins was not observed in anoikis resistant cancer cells (below and supplemental Fig. S3). Taken together, these results suggest that Ad-E1A12 infection of epithelial cancer cells triggers spontaneous matrix detachment, resulting in downregulation of antiapoptotic proteins and eventual apoptosis.

### Distinct mechanisms of AKT activation upon Ad-E1A12 infection in epithelial and mesenchymal cancer cells

**Figure 3:**
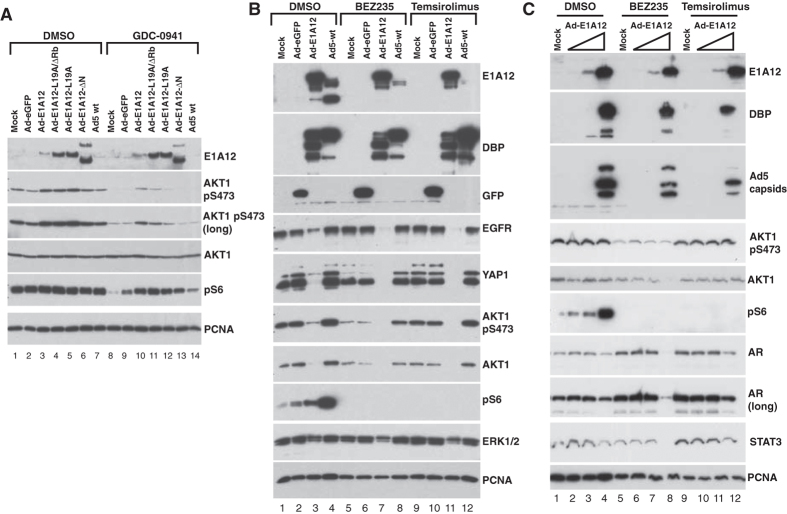
PI3K inhibition suppresses Ad-E1A12-induced phosphorylation of AKT1 but not S6 in epithelial cancer cells. (**A**) MDA-MB-468 cells were uninfected or infected with the indicated viruses (100 vps/cell). DMSO or an equal volume of GDC-0941 was added to 0.1 μM 2 h after adding virus in the indicated lanes. At 24 hpi, cells were lysed for Western blotting with antibodies against the indicated proteins. (**B**) Suppression of cell survival pathways in Ad-E1A12-infected MDA-MB-468 cells. MDA-MB-468 cells were treated as in panel A. The indicated drugs were added to 0.1 μM. At 48 hpi, cells were lysed for Western blotting with antibodies against the indicated proteins. (**C**) LNCaP cells were uninfected (mock) or infected with increasing amount of Ad-E1A12 (10, 100, 1,000 vps/cell, from left to right in each group). Drugs were added as in panel (**B**). Cells were harvested at 48hpi for Western blotting.

Since AKT activation is critical for cell survival, we assessed AKT phosphorylation status in cells infected with Ad-E1A12. Under normal cell culture conditions, both mTOR and AKT pathways were highly active in MDA-MB-468 cells (lane 1 in Fig. 3A). Infection with Ad-E1A12 or viruses with mutant E1A12 moderately increased AKT1 pS473 levels at 24hpi (Fig. 3A). Treatment of the infected cells with the pan-PI3K inhibitor GDC-0941 markedly reduced AKT1 pS473 levels, suggesting that PI3K signaling is largely responsible for AKT1 phosphorylation in MDA-MB-468. GDC-0941 treatment also dramatically suppressed the phosphorylation of ribosomal protein S6 (pS6) in uninfected MDA-MB-468 cells (Fig. 3A lane 8), indicating that mTOR activation is mediated through the upstream PI3K-AKT signaling in MDA-MB-468 cells. However, the pS6 levels were maintained in virus-infected cells even in the presence of GDC-0941, especially in cells infected with Ad-E1A12 or viruses expressing E1A12 mutants (Fig. 3A). This is likely due to E1A-activated expression of two viral proteins E4-ORF1 and E4-ORF4 that potently activate PI3K-AKT and mTOR pathways respectively^28^,^29^,^30^. At 48hpi when MDA-MB-468 cells already underwent apoptosis (Fig. 1), there was a dramatic decrease in total AKT1 levels and AKT activation as measured by AKT1 phosphorylation at Ser473 (Fig. 3B). Concomitantly, EGFR and YAP1 were degraded in Ad-E1A12-infected cells. Thus, MDA-MB-468 cells infected with Ad-E1A12 were unable to sustain AKT activation compared to cells infected with control Ad-eGFP or wt Ad5 (Fig. 3B). Surprisingly, mTOR signaling as measured with the pS6 levels was increased in cells infected with Ad-E1A12 compared to those in uninfected or Ad-eGFP-infected cells, and wt Ad5 elicited stronger mTOR signaling (Fig. 3B lane 4). Treatment of infected cells with dual PI3K/mTOR inhibitor NVP-BEZ235 (BEZ235) or the allosteric mTORC1 inhibitor temsirolimus exacerbated the degradation of EGFR, AKT and YAP1 (Fig. 3B) as well as cell death (data not shown). Analogous to MDA-MB-468 cells, AKT is constitutively active in the LNCaP cell line, and viral infection did not significantly impact AKT activation, although moderate downregulation of total AKT1 was observed (Fig. 3C). Furthermore, mTOR signaling was markedly activated in Ad-E1A12-infected LNCaP cells in a dose-dependent manner. Treatment of the infected LNCaP cells with BEZ235 but not temsirolimus resulted in a dramatic reduction of AKT1 pS473 levels and the degradation of AKT1 in addition to the androgen receptor (AR) and STAT3 (Fig. 3C), suggesting that the inhibition of PI3K by BEZ235 augments Ad-E1A12-mediated apoptosis. Taken together, these data indicate that the canonical PI3K-AKT-mTOR signaling pathway is constitutively active in epithelial cancer cells and that this pathway cannot be sustained, when AKT1 and other proteins critically involved in cell survival are degraded upon Ad-E1A12 infection.

**Figure 4:**
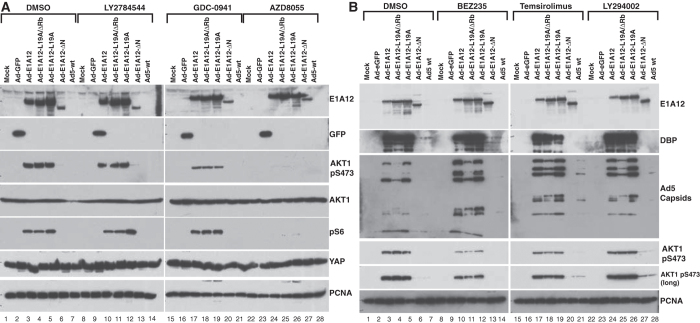
PI3K-independent hyperactivation of AKT and mTOR signaling by Ad-E1A12. MDA-MB-231 cells were infected with the indicated viruses (100 vps/cell). The cells were then exposed to the indicated inhibitors at 0.1 μM (LY294002 was at 1 μM) at 2hpi. These inhibitors are specific to JAK2 (LY2784544), all isoforms of PI3K (GDC-0941), mTORC1 and mTORC2 (AZD8055), PI3K/mTOR1/mTORC2 (BEZ235), mTORC1 (temsirolimus), and PI3K (LY294002). The cells were lysed at 24 hpi (**A**) or 48hpi (**B**). The lysates were subjected to Western blotting analysis with antibodies against the indicated proteins.

**Figure 5:**
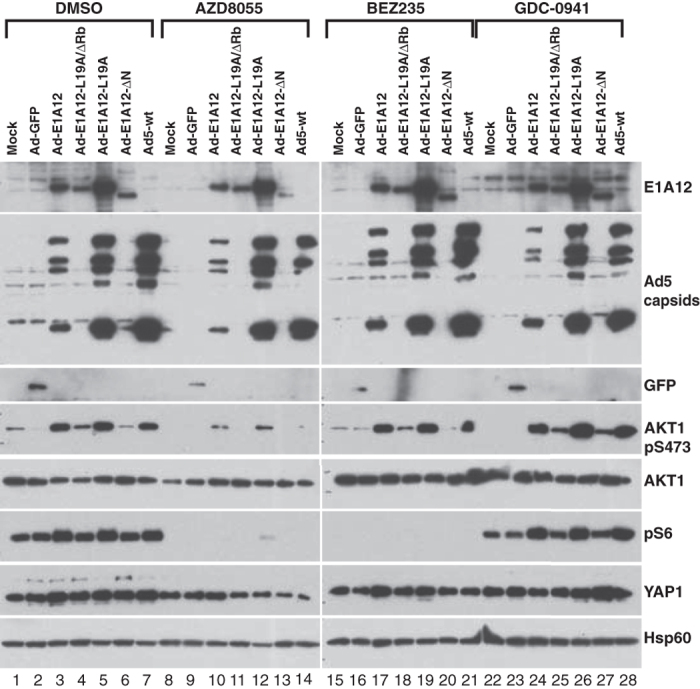
Ad-E1A12 activates PI3K-independent AKT and mTOR signaling in PC-3 cells. PC-3 cells were infected with the indicated viruses (100 vps/cell). The infected cells were treated with DMSO or an indicated inhibitor at 0.1 μM. Cells were lysed at 48hpi for Western blotting analysis with antibodies against the indicated proteins.

Infection of mesenchymal MDA-MB-231 cancer cells with Ad-E1A12 also resulted in dramatic activation of AKT and mTOR signaling (Fig. 4). However, compared to infected MDA-MB-468 cells, YAP1 was not degraded and AKT hyperactivation was sustained in MDA-MB-231 cells in Ad-E1A12-infected cells (Fig. 4). Furthermore, Ad-E1A12 infection did not induce cell death in MDA-MB-231 cells, up to 96h post-infection (see below). This sustained AKT signaling might account for the resistance of MDA-MB-231 to apoptosis upon Ad-E1A12 infection. Notably, PI3K inhibition with GDC-0941 attenuated AKT signaling, but substantial levels of phosphorylated AKT1 remained (Fig. 4A lanes 17–19). Likewise, treatment of the infected cells with another PI3K inhibitor LY294002 (Fig. 4B lanes 24–26) or the JAK2 kinase inhibitor LY2784544 (lanes 10–12 in Fig. 4A) did not affect AKT activation. In contrast, the highly potent and selective mTORC1/2 inhibitor AZD8055 almost completely suppressed AKT and mTOR signaling (Fig. 4A lanes 24–26). Of note, the exposure of the infected cells to the PI3K/mTOR dual inhibitor BEZ235 only partially inhibited AKT activation (Fig. 4B lanes 10–12; also see Fig. 5). These results suggest that AKT activation is largely mediated through mTORC2 upon Ad-E1A12 infection in mesenchymal MDA-MB-231 cells in contrast to the reliance of the upstream PI3K signaling for AKT activation in epithelial MDA-MB-468 and LNCaP cells (Fig. 3).

We also tested effects of mutations in E1A12 on apoptosis and signaling pathways. Deletion of the N-terminal 29 amino acid residues of E1A12 (E1A12-ΔN) completely abolished the ability of the recombinant virus to induce apoptosis (data not shown) and to activate AKT and mTOR signaling in MDA-MB-231 cells (Fig. 4). Other mutations (L19A and L19A/ΔRb) had no or only very slight effects (Fig. 4). E1A12-ΔN was also defective in activating viral early (DBP) and late (Ad5 capsid) gene expression (Fig. 4B). Since the E1A N-terminal domain is required for binding p300/CBP^21^, these observations indicate that these transcriptional coactivators are critical for E1A-mediated gene expression. Our data further indicate that induction of apoptosis and cellular signaling by Ad-E1A12 is mediated through potent activation of gene expression by E1A12.

Similarly, hyperactivation of AKT and mTOR signaling also occurred in mesenchymal PC-3 cells upon Ad-E1A12 infection (Fig. 5). The pan-PI3K inhibitor GDC-0941 had no effects on AKT and mTOR signaling. However, the mTORC1/2 inhibitor AZD8055 markedly suppressed both pathways. These data suggest again that, in mesenchymal cancer cells, AKT activation by Ad-E1A12 is largely PI3K-independent, but depends on mTORC2. Notably, substantial AKT signaling persisted in the presence of BEZ235 in PC-3 cells infected with Ad-E1A12, Ad-E1A12-L19A, or Ad5 (lanes 17, 19 and 21 in Fig. 5). This could be due to a feedback effect as a result of the inhibition of PI3K by BEZ235, because the PI3K inhibitor GDC-0941 also seemed to intensify virus-induced AKT1 and S6 phosphorylation (lanes 24, 26 and 28 in Fig. 5). Interestingly, the E1A12 mutant defective of Rb-binding (Ad-E1A12-L19A/ΔRb) was largely inactive in inducing both AKT and mTOR signaling (Fig. 5). Like the Ad-E1A-ΔN virus, this mutant virus was also defective in inducing viral gene expression (see the Ad5 capsid panel in Fig. 5), suggesting once again that E1A12-mediated gene activation is critical to activating cellular signaling pathways. Consistent with the notion that PC-3 cells are resistant to Ad-E1A12-induced apoptosis, AKT1 and YAP1 were not degraded in Ad-E1A12-infected PC-3 cells (Fig. 5; also see Fig. 2). In fact, a slight increase of the YAP1 levels can be seen in virus-infected PC-3 cells. Thus, apoptosis-resistant cancer cells activate AKT through mTORC2 independently of PI3K.

### *In vivo* effects of Ad-E1A12 on tumor growth of MDA-MB-468 and MDA-MB-231 xenografts

**Figure 6:**
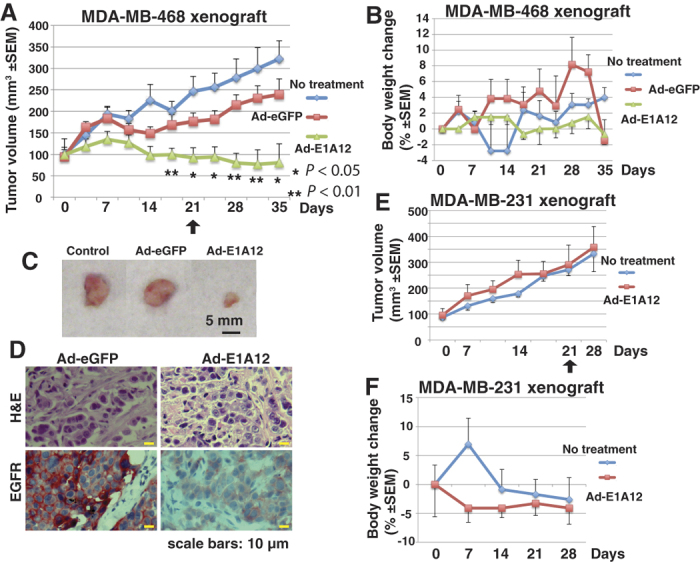
Ad-E1A12 suppresses tumor growth of the MDA-MB-468 xenograft but not the MDA-MB-231 xenograft. Xenografts were established by injecting MDA-MB-468 cells with Matrigel or MDA-MB-231 cells in the flanks of female NU/NU mice. Two injections of viruses (1 × 10^9^ vps per injection) per week for three weeks were done. The treatment endpoint is indicated with an arrow. Tumor volumes (**A**,**E**) and percent body weight changes (**B** and **F**) of the tumor-bearing mice are shown. Images of representative tumors (**C**) and histochemical staining (**D**) of MDA-MB-468 xenograft tumors are also shown. Each treatment group had 5 mice.

Data presented above demonstrate markedly different sensitivities of cancer cells to Ad-E1A12-induced apoptosis. To assess whether this would also apply *in vivo*, we have treated tumors of Ad-E1A12-sensitive MDA-MB-468 and resistant MDA-MB-231 xenografts in mice through intratumoral injection. As shown in Fig. 6A, the MDA-MB-468 tumors underwent a marked regression after receiving a total of six doses of Ad-E1A12 injections, whereas Ad-GFP had no effects. Consistent with our *in vitro* observations, EGFR was markedly downregulated in tumors treated with Ad-E1A12 compared to those injected with Ad-GFP (Fig. 6D). The treatment regimen was well tolerated, as the body weight change of the tumor-bearing mice did not exceed 10% during treatment (Fig. 6B). In contrast, Ad-E1A12 injection exerted no effects on MDA-MB-231 xenografts (Fig. 6E). Thus, the sensitivity or resistance to apoptosis is maintained *in vivo*.

### Expression of the miR-200 family sensitizes MDA-MB-231 cells to Ad-E1A12-mediated cell death

**Figure 7:**
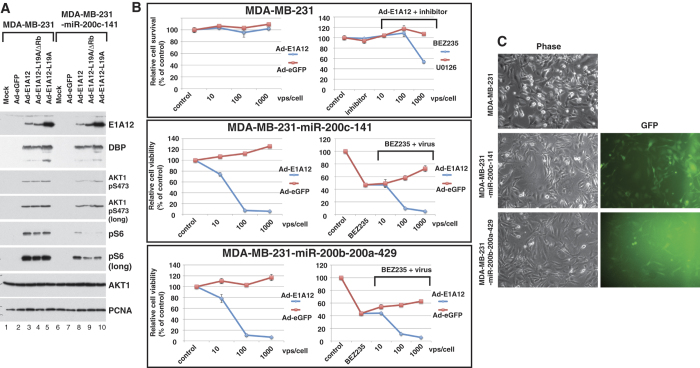
Ectopic expression of miR-200 inhibits AKT/mTOR signaling and restores the sensitivity to Ad-E1A12-induced cell death. MDA-MB-231 cells were stably transduced with a lentiviral vector carrying an expression cassette of the *miR-200c-141-GFP* or the *miR-200b-200a-429-GFP* cluster. (**A**) the transduced cells were infected with the indicated viruses and harvested at 48hpi for Western blotting. (**B**) the transduced cells were infected with an indicated adenovirus in the absence or the presence of an indicated inhibitor (0.1 μM). Cell viability was determined at 96 hpi. The relative cell viability vs. control is plotted against the viral dosage (average ± SEM, n = 3). (**C**) representative images of the parental and transduced MDA-MB-231 cells are shown. The corresponding micrographs of GFP field for the transduced cells are also shown.

The miR-200 family of microRNAs is expressed in epithelial cancer cells but downregulated in mesenchymal cancer cells^10^,^31^,^32^. Ectopic expression of the miR-200 family in mesenchymal cancer cells restores E-cadherin expression and other epithelial phenotypes as well as suppresses xenograft tumor growth *in vivo*^10^,^31^,^33^. To assess whether enforced expression of miR-200 in mesenchymal cancer cells would affect sensitivity to Ad-E1A12-mediated apoptosis, we stably expressed the miR-200 family in MDA-MB-231 cells. Compared to the parental cells, infection of the MDA-MB-231 cells with miR-200 expression with Ad-E1A12 or a virus with a mutant E1A12 attenuated AKT1 phosphorylation and markedly suppressed S6 phosphorylation (Fig. 7A). Ectopic expression of the miR-200b-200a-429 or the miR-200c-141 cluster dramatically sensitized MDA-MB-231 cells to Ad-E1A12-induced cell death, whereas the parental cells were highly resistant (Fig. 7B). Notably, treatment of Ad-E1A12-infected parental MDA-MB-231 cells with BEZ235 but not the MEK inhibitor U0126 impaired cell viability (Fig. 7B). Interestingly, BEZ235 treatment did not further sensitize MDA-MB-231 cells with ectopic miR-200 expression to Ad-E1A12 (Fig. 7B). These observations provide the first evidence that the miR-200 family inhibits mTOR signaling and that the reversion of mesenchymal cancer cells to epithelial phenotypes increases their sensitivity to Ad-E1A12-triggered apoptosis.

## Discussion

This study presents evidence for the first time that distinct cell survival signaling mechanisms in epithelial and mesenchymal cancer cells seem to determine sensitivity to virus-induced apoptosis. Our data indicate that Ad-E1A12 infection resulted in hyperactive AKT phosphorylation in diverse cancer cell lines. Under conventional cell culture conditions, the levels of AKT phosphorylation are much higher in both MDA-MB-468 and LNCaP cells (Fig. 3) than in MDA-MB-231 and PC-3 cells (Figs 4 and 5). In epithelial MDA-MB-468 and LNCaP cells, the PI3K-AKT axis seems largely responsible for AKT phosphorylation, as the inhibition of PI3K with GDC-0941 or BEZ235 profoundly suppressed AKT phosphorylation (Fig. 3). In contrast, PI3K inhibition had little effects on AKT phosphorylation in MDA-MB-231 (Fig. 4) and PC-3 cells (Fig. 5) after Ad-E1A12 infection. However, the ATP-competitive mTOR inhibitor AZD8055 that inhibits both mTORC1 and mTORC2 essentially abolished AKT phosphorylation in Ad-E1A12-infected MDA-MB-231 and PC-3 cells (Figs 4 and 5). Interestingly, PI3K inhibition with GDC-0941 appears to exert a small but noticeable enhancement of AKT phosphorylation in PC-3 cells (Fig. 5). This apparent feedback effect could explain why BEZ235 (a dual PI3K and mTOR1/2 inhibitor) was less effective than AZD8055, which only inhibits mTORC1/2, in suppressing AKT phosphorylation in mesenchymal cells (Figs 4 and 5).

The importance of the PI3K-AKT signaling for epithelial cell survival is well documented. The viability of ECM-deprived MCF-10A cells (immortalized but nonmalignant human mammary epithelial cells) can be rescued by restoring PI3K-AKT signaling, for example by ERBB2-mediated stabilization of EGFR^4^. As noted above, the PI3K-AKT pathway is constitutively active in the epithelial MDA-MB-468 and LNCaP cells. Their detachment from ECM resulted in AKT degradation and impaired viability. In contrast, sustained AKT phosphorylation was maintained in the mesenchymal MDA-MB-231 and PC-3 cells infected with Ad-E1A12, not through the canonical PI3K-AKT axis, but rather through the mTORC2-AKT pathway independently of PI3K. This observation suggests that epithelial cancer cells rely heavily on RTK-PI3K-AKT signaling for survival, whereas mesenchymal cancer cells maintain survival through different mechanisms, with the mTORC2-AKT signaling pathway representing one probable mechanism. Consistent with this hypothesis, MDA-MB-468 cells are much more sensitive to pharmacologic inhibition of all PI3K isoforms than MDA-MB-231 cells^34^,^35^. Likewise, LNCaP cells are at least 7.5-fold more sensitive to PI3K inhibition with GDC-0941 or AZD6482 than PC-3 cells (http://www.cancerrxgene.org/).

Acquired resistance to apoptosis is critical for epithelial cancer cells to survive and metastasize in distant organs^5^,^6^. The importance of the mTORC2-AKT axis for the survival of mesenchymal cancer cells in the presence of strong apoptotic stimuli suggests that this signaling pathway might play a major role in tumor metastatic progression. Indeed, mounting evidence suggests that mTORC2 signaling promotes metastatic progression^36^,^37^,^38^,^39^ and resistance to chemotherapy^40^. For example, mTORC2 blocks Cbl-mediated ubiquitination and degradation of the short form of c-FLIP, thereby suppressing TRAIL-induced and death receptor 4 (DR4)-mediated apoptosis^41^. TRAIL is a member of the tumor necrosis factor (TNF) superfamily. Interestingly, the detachment of PC-3 results in the upregulation of TNFα^42^, which activates AKT signaling and cell survival in PC3 cells^43^. Thus, mTORC2 activation may be particularly important for cell survival of mesenchymal cancer cells.

The cancer-specific metabolic shift may also rely on active mTORC2 signaling. ECM detachment of epithelial cells results in ATP depletion, reduced glucose uptake, and increased levels of ROS (reactive oxygen species)^4^. Reduced ATP levels impair mTORC2 kinase activity, which is highly sensitive to ATP depletion^44^. Furthermore, mTORC2 activity promotes glucose uptake^45^,^46^. Consistently, liver-specific Rictor knockout reduces glucose uptake, and constitutively active AKT was sufficient to restore normal glucose tolerance in hepatocytes^46^,^47^,^48^. Thus, a positive feedback loop may exist between glucose uptake, cellular ATP levels and mTORC2 kinase activity. These observations indicate that the ability of cancer cells to maintain mTORC2 activity may be critical for their survival in a nonnative microenvironment as well as for maintaining glycolysis (the Warburg effect) for anabolic biosynthesis required for rapid proliferation^49^. Mutations of the components of the mTORC2 complex such as SIN1 are identified in cancers^50^. Such mutations promote sustained AKT activation and tumor growth^51^. Furthermore, the development of prostate cancer as a result of PTEN loss depends on mTORC2 activity^52^.

Whereas Ad-E1A12 infection of MDA-MB-231 cells results in marked activation of ATK1 and S6 phosphorylation *in vitro* (Fig. 4), intratumoral injection of Ad-E1A12 viral particles only moderately enhanced tumor growth in the early time points, but this enhancement was lost in the late time points (Fig. 6E). It is possible that at early time points when tumors are small, the injected virus could reach most parts of the tumors, providing a boost to tumor growth through activating the AKT/mTOR pathway. However, at the late time points when tumors become larger, the penetration of injected virus is limited and hence its effects on tumor growth is diminished. Regardless, the virus-induced activation of AKT/mTOR signaling *in vivo* seems temporary and therefore its impact on tumor growth is probably quite limited.

Earlier studies demonstrate that Ad5 E1A 243R sensitizes cells to anoikis^17^,^18^. Induction of genes that confer epithelial phenotype such as CDH1 (encoding E-cadherin) by E1A seems to play a role in anoikis sensitization^17^,^18^. Herein we have used a viral vector for expressing the large E1A variant from the oncogenic Ad12 species (Ad-E1A12). Our data show that cell detachment is not required for this virus to trigger cell death; but cell detachment upon Ad-E1A12 infection results in marked downregulation of various important players involved in cell survival such as EGFR, AKT1 and YAP1 (Figs 2 and 3), leading to rapid demise of infected epithelial cancer cells. The data herein also indicate that the ability of E1A12 to activate gene expression is critical for cell detachment and death, as mutants that could not activate gene expression were defective in triggering cell detachment and apoptosis (Figs 3, 4, 5). We should point out that there are fundamental differences between Ad-E1A12 and Ad5 E1A 243R. First, the large E1A isoform used in this study contains the conserved region 3 (CR3) that is absent in the small E1A isoform such as the Ad5 E1A 243R. CR3 encodes a zinc-finger that interacts with the MED23 subunit of the Mediator complex and is a powerful activator of transcription^53^. Second, E1A12 is expressed from a viral vector that is replication-competent (Fig. 1). Thus, Ad-E1A12 infection is likely to exert a much broader impact on gene expression than E1A 243R. Further studies are needed to fully understand how Ad-E1A12 triggers spontaneous detachment of epithelial cancer cells and apoptosis.

In conclusion, our study uncovers distinct cell survival signaling mechanisms in anoikis-sensitive and resistant cancer cells. Cancer cells of epithelial phenotypes depend on PI3K-AKT signaling for survival, but this cannot be sustained due to detachment-induced AKT degradation. Mesenchymal cancer cells maintain AKT activation through PI3K-independent mTORC2 signaling in response to Ad-E1A12 infection. Our results suggest that targeting mTORC2-AKT signaling might offer therapeutic opportunities for combatting metastases. Although selective and ATP-competitive inhibitors of mTOR could be used, inhibitors that only suppress the mTORC2 signaling might be more effective for eliminating metastases, as mTORC1 inhibition with the allosteric inhibitor RAD001 seems to promote metastasis^54^.

## Materials and Methods

### Materials, cell culture and viruses

Small-molecule inhibitors were obtained from Selleck. The cell lines used in this study were from ATCC. To express the miR-200 family, MDA-MB-231 cells were stably transduced with lentiviruses carrying an expression cassette for the *miR-200b-200a-429* or the *miR-200c-141* cluster as described^10^. Ad-E1A12 (Fig. 1) was constructed by inserting an expressing cassette of the red fluorescent protein (RFP) and Ad12 E1A 266R cDNAs separated with an internal ribosomal entry site (IRES) under the control of the cytomegalovirus immediate-early (CMV *IE*) promoter in the *E1* region of an *E1* and *E3*-deleted Ad vector (pAdEasy-1, Stratagene). Mutations in E1A12 were made using the QuickChange kit (Stratagene). Viruses carrying a mutated E1A12 were constructed similarly. The viruses were packaged in 293 cells, amplified, purified and titered by ViraQuest, Inc. Wild-type Ad5 and Ad12 viruses were obtained from ATCC and purified high-titer viral stocks were produced by ViraQuest, Inc. Viral DNAs isolated from infected cells were used for quantitative real-time PCR with the primer pair of 5′-GAGGAGGAGGACATGATGGA-3′ and 5′-CTGAGGAGCGGAGGTTGTAG-3′. Relative viral genome abundance was determined by the differential cycle threshold (ΔC_t_) between the C_t_ values of viral DNA isolated from cells infected in replication-defective Ad-eGFP and those infected with Ad-E1A12 or wt Ad5. The experiments were repeated for at least three times with triplicate PCR reactions each time.

### Flow cytometry, cell viability assays and Western blotting

Flow cytometry of control and virus-infected cells was done as described previously^55^. The viability of cells infected with viruses or treated with an inhibitor was determined using the CellTiter-Glo kit (Promega) as published^56^. The EC_50_ of Ad-E1A12 against various cancer cell lines was determined by fitting viability data using nonlinear regression as implemented in the Prism 6 software. For assessing effects of cell detachment on Ad-E1A12-induced cell death, MDA-MB-468 cells were mock treated or infected with viruses. At 4 h post infection, cells were trypsinized and reseeded in a regular 96-well plate or an ultra-low attachment 96-well plate. Viability was assayed at 24 h and 48 h post infection. For Western blotting, cells in multiple-well plates were lysed and processed as described previously^57^. Antibodies used for Western blotting detection included those against Ad12 E1A, Ad5 DBP (B6–8), and Ad5 capsids as previously described^57^. In addition, an custom-made anti-Ad12 E1A polyclonal antibody by YenZym Antibodies, LLC was also used for detecting Ad12 E1A. Commercial antibodies against various proteins were purchased from Santa Cruz Biotechnology (Ad5 E1A: sc-25, p53: DO-1, EGFR: sc-03, STAT3: sc-482, Bcl-xL: sc-634), Cell Signaling Technology (Survivin: #2808, S6 pSer235/236: #2211), BD Bioscience (Hsp60: H99020), Covance (GFP: MMS-118P), Sigma-Aldrich (Erk1/2: M5670), and Epitomics (Akt1: 1085–1, AKT1 pSer473: 2118-1, and PCNA: 2714-1).

### Quantitative real-time RT-PCR

MDA-MB-468 cells were uninfected or infected with Ad-eGFP, Ad-E1A12 or wt Ad5 (1,000 vps/cell). At 24 h post-infection, RNAs were extracted using the RNeasy kit (Qiagen). cDNAs were synthesized from total RNAs using MultiScribe Reverse transcriptase kit (Applied Biosystems). The cDNAs were used as templates for real-time PCR with SYBR-green detection. Quantification was done as described above. The PCR primers are: YAP1 (5′-CAGCAACTGCAGATGGAGAA-3′ and 5′-TGGATTTTGAGTCCCACCAT-3′); BIRC5 (5′- GCCCAGTGTTTCTTCTGCTT-3′ and 5′-TCTCCGCAGTTTCCTCAAAT-3′); STAT3 (5′- CAGTCAGTGACCAGGCAGAA-3′ and 5′-CGTACTCCATCGCTGACAAA-3′); CTGF (5′- GCAGGCTAGAGAAGCAGAGC-3′ and 5′- TGGAGATTTTGGGAGTACGG-3′); and ACTB (5′- GCTCCTCCTGAGCGCAAGTACTC-3′ and 5′- GTGGACAGCGAGGCCAGGAT-3′).

### Animal studies

Five million cancer cells were suspended in 100 μl of PBS (for MDA-MB-231 cells) or in 50 μl of PBS mixed with 50 μl of Matrigel (for MDA-MB-468 cells). The mixture was injected into the flank of a female NU/NU mouse (5–7 week old, Charles River). Palpable tumors developed in two to three weeks. Tumor volume was determined as described^56^. When tumor volume reached approximately 100 mm^3^, mice were randomized into treatment groups (n = 5). These mice either received no treatment, or two weekly intratumoral injections of Ad-eGFP or Ad-E1A12 at the dose of 1 × 10^9^ vps in 100 μl of PBS per injection twice weekly for three weeks. Tumor-bearing mice were monitored for three additional weeks after treatment termination. The mice were then euthanized and their tumors dissected and sectioned for hematoxylin and eosin staining and immunohistochemical detection of EGFR with anti-EGFR antibody (Santa Cruz Biotechnology, sc-03). Data are shown as means ± SEM (standard error of the means). The two-tailed Student’s t test was used to compare differences between treatment groups. The differences were considered statistically significant if *P* < 0.05. The animal protocol was approved by the University of Florida IACUC. All experiments were performed in accordance with relevant guidelines and regulations.

## Additional Information

**How to cite this article**: Wang, Y. *et al.* Intrinsic cellular signaling mechanisms determine the sensitivity of cancer cells to virus-induced apoptosis. *Sci. Rep.* **6**, 37213; doi: 10.1038/srep37213 (2016).

**Publisher’s note**: Springer Nature remains neutral with regard to jurisdictional claims in published maps and institutional affiliations.

## Supplementary Material

Supplementary Information

## Figures and Tables

**Table 1:** EC_50_ values of Ad-E1A12 against various cancer cell lines.

Cell line	EC_50_ (vps per cell)
MDA-MB-468	594
MDA-MB-231	>1,000*
MDA-MB-436	306
MCF7	511
PANC-1	>1,000*
MIA PaCa-2	>1,000*
BxPC3	8
HPAC	7
LNCaP	665
PC-3	>1000*
HCT116	325

^*^Note: EC_50_ was not reached at the highest dose (1,000 vps/cell).
