# Supplementary material for: Intrinsic cellular signaling mechanisms determine the sensitivity of cancer cells to virus-induced apoptosis
Source: Sci Rep. 2016 Nov 16;6:37213. doi: 10.1038/srep37213 (PMC5111159; doi:10.1038/srep37213)
Supplement: Supplementary Information [file srep37213-s1.pdf]

Intrinsic cellular signaling mechanisms determine the sensitivity of cancer cells to virus-induced apoptosis

Yunfei Wang, Dawei Li, Jian Luo, Guimei Tian, Lisa Y. Zhao, and Daiqing Liao

Supplementary Information

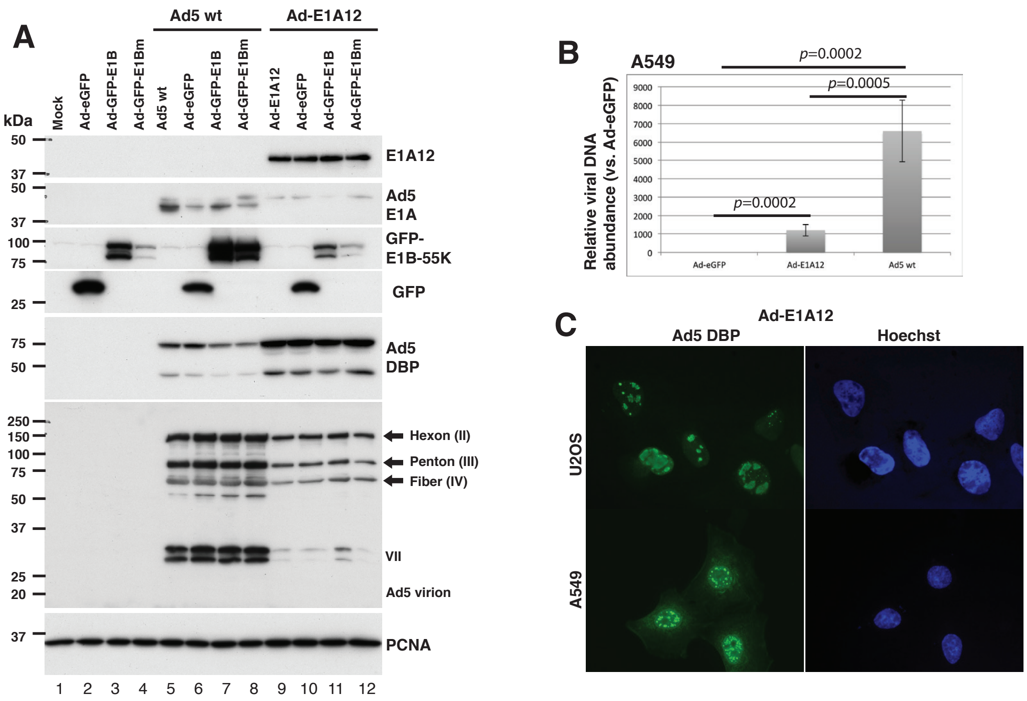

Figure S1. Replication of Ad-E1A12 in various cancer cell lines. A, A549 cells were infected with the indicated combinations of adenoviruses and the infected cells were harvested at 48hpi for Western blotting. Viruses expressing the E1B 55-kDa (Ad-GFP-E1B) or its mutant with the S476/477A mutations (Ad-GFP-E1Bm) were superinfected with wt Ad5 or Ad-E1A12 to assess whether E1B-55-kDa expression would enhance Ad reproduction. B, genome replication of wt Ad5 and Ad-E1A12 in A549 cells. The relative abundance of viral genomic DNA at 48hpi was determined using quantitative real-time PCR. The P-value was determined with Student's t-test. C, viral replication centers in Ad-E1A12-infected cells. The indicated cell lines were infected with Ad-E1A12 and the cells were fixed for immunostaining with antibody against DBP and counter stained with the Hoechst dye.

Wang et al., Supplemental Figure S2

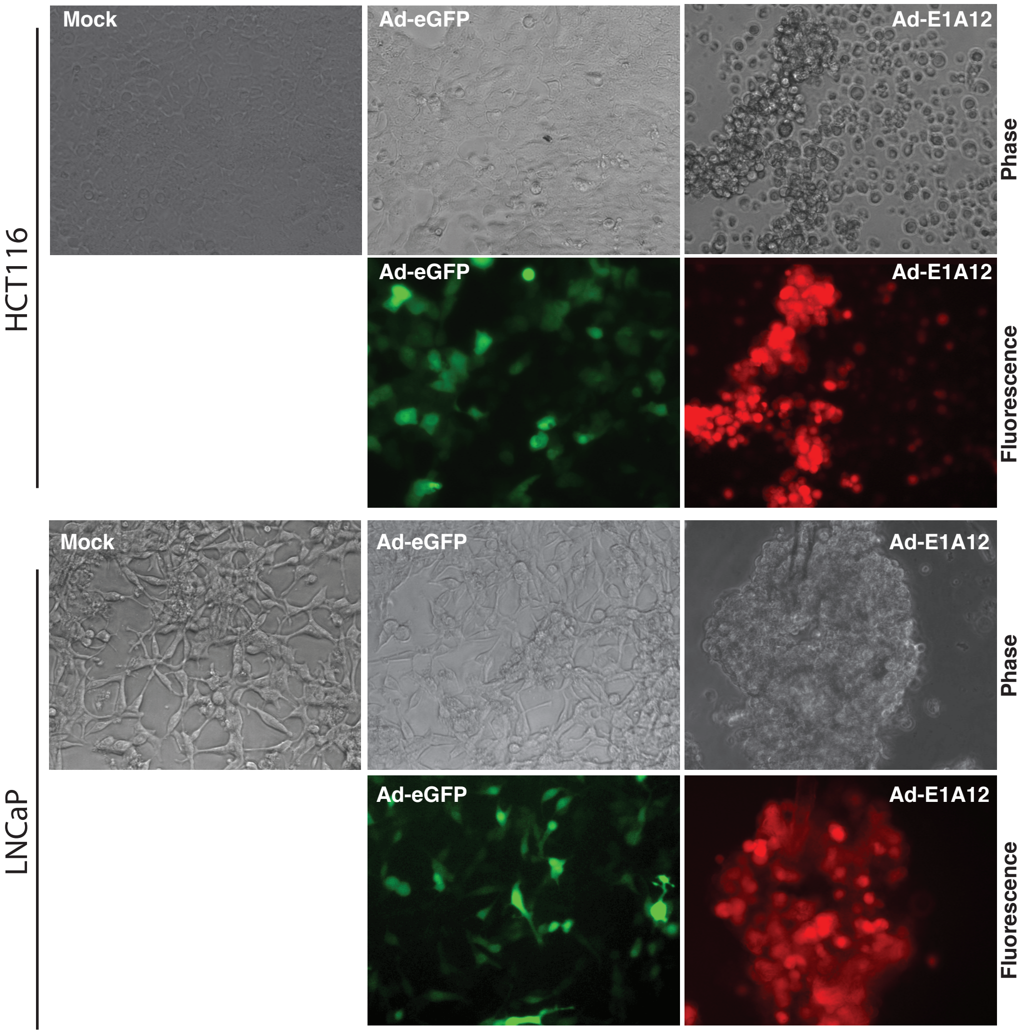

Figure S2. Ad-E1A12 infection triggers detachment of HCT116 and LNCaP cells. The cells were infected with Ad-eGFP or Ad-E1A12. The infected cells were photographed at 48hpi. Representative phase and fluorescence images of the cell cultures are shown. Note the detachment of Ad-E1A12-infected cells.

Wang et al., Supplemental Figure S3

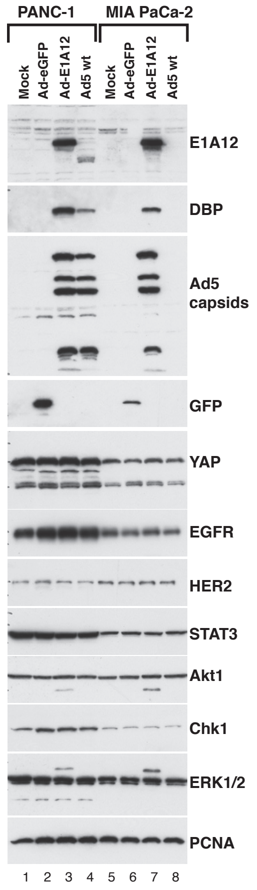

Figure S3. Ad-E1A12 infection does not induce anoikis in the PANC-1 and MIA PaCa-2 pancreatic cancer cell lines. The cells lines were infected with an indicated virus (100 vps/cell). The cells were harvested at 48hpi for Western blotting with antibodies against the indicated proteins.
